# Development of Zika Virus Mini-Replicon Based Single-Round Infectious Particles as Gene Delivery Vehicles

**DOI:** 10.3390/v15081762

**Published:** 2023-08-18

**Authors:** Joh-Sin Wu, Ju-Ying Kan, Hsueh-Chou Lai, Cheng-Wen Lin

**Affiliations:** 1 The PhD Program for Health Science and Industry, China Medical University, Taichung 404333, Taiwan; u108310001@cmu.edu.tw; 2 Department of Medical Laboratory Science and Biotechnology, China Medical University, No. 91, Hsueh-Shih Road, Taichung 404333, Taiwan; u110311201@cmu.edu.tw; 3 The PhD Program of Biotechnology and Biomedical Industry, China Medical University, Taichung 404333, Taiwan; 4 Division of Hepato-Gastroenterology, Department of Internal Medicine, China Medical University Hospital, Taichung 404332, Taiwan; 003145@tool.caaumed.org.tw; 5 Drug Development Center, China Medical University, Taichung 404333, Taiwan; 6 Department of Medical Laboratory Science and Biotechnology, Asia University, Wufeng, Taichung 413305, Taiwan

**Keywords:** Zika virus, single-round infectious particle, mini-replicon, reporter gene, hACE2, gene delivery vehicle

## Abstract

Zika virus (ZIKV) is a type of RNA virus that belongs to the Flaviviridae family. We have reported the construction of a DNA-launched replicon of the Asian-lineage Natal RGN strain and the production of single-round infectious particles (SRIPs) via the combination of prM/E virus-like particles with the replicon. The main objective of the study was to engineer the ZIKV replicon as mammalian expression vectors and evaluate the potential of ZIKV mini-replicon-based SRIPs as delivery vehicles for heterologous gene expression in vitro and in vivo. The mini-replicons contained various genetic elements, including NS4B, an NS5 methyltransferase (MTase) domain, and an NS5 RNA-dependent RNA polymerase (RdRp) domain. Among these mini-replicons, only ZIKV mini-replicons 2 and 3, which contained the full NS5 and NS4B-NS5 genetic elements, respectively, exhibited the expression of reporters (green fluorescent protein (GFP) and cyan fluorescent protein–yellow fluorescent fusion protein (CYP)) and generated self-replicating RNAs. When the mini-replicons were transfected into the cells expressing ZIKV prM/E, this led to the production of ZIKV mini-replicon-based SRIPs. ZIKV mini-replicon 3 SRIPs showed a significantly higher yield titer and a greater abundance of self-replicating replicon RNAs when compared to ZIKV mini-replicon 2 SRIPs. Additionally, there were disparities in the dynamics of CYP expression and cytotoxic effects observed in various infected cell types between ZIKV mini-replicon 2-CYP and 3-CYP SRIPs. In particular, ZIKV mini-replicon 3-CYP SRIPs led to a substantial decrease in the survival rates of infected cells at a MOI of 2. An in vivo gene expression assay indicated that hACE2 expression was detected in the lung and brain tissues of mice following the intravenous administration of ZIKV mini-replicon 3-hACE2 SRIPs. Overall, this study highlights the potential of ZIKV mini-replicon-based SRIPs as promising vehicles for gene delivery applications in vitro and in vivo.

## 1. Introduction

Flaviviruses, including Zika virus (ZIKV), dengue virus, Japanese encephalitis virus (JEV), West Nile virus, and yellow fever virus, belong to a group of RNA viruses [1,2]. The flaviviral genome encodes a polyprotein consisting of ten proteins, including three structural proteins (C, prM, and E) and seven non-structural proteins (NS1, NS2A, NS2B, NS3, NS4A, NS4B, and NS5). The E protein is positioned within the lipid membrane surrounding the capsid and plays a crucial role in binding to receptors and attachment factors on the surface of cells [3]. The NS2B-NS3 protease facilitates the proteolytic processing of non-structural proteins. Among these non-structural proteins, the RNA helicase located at the C-terminus of NS3, the methyltransferase (MTase) at the N-terminus of NS5, and the RNA-dependent RNA polymerase (RdRp) at the C-terminus of NS5 are responsible for the synthesis of viral RNA genomes [4]. Transmembrane proteins, specifically NS4A and NS4B, play crucial roles within the replication complex on the endoplasmic reticulum membrane. They facilitate the close interaction between enzymes like NS3/NS5 and viral RNA, which is of paramount importance for the successful replication of the virus.

Flaviviral single-stranded, positive-sense RNA genomes have been manipulated to create viral replicons [5,6,7,8,9,10,11]. Flavivirus replicons are designed by removing the structural protein genes necessary for viral assembly while retaining the essential elements and non-structural protein genes required for self-replication. This design offers advantages as a vector system, including efficient gene expression, self-replication capability, and compatibility with a broad range of hosts. The self-replication of flavivirus replicons allows for the amplification of the input template in the host cell, leading to the increased production of encoded heterogeneous proteins. DNA-launched replicons provide greater convenience compared to RNA-launched replicons, as the latter require the production of viral RNA subgenomes using in vitro transcription assays [12]. In contrast, DNA-launched replicons consist of a partial genome that includes cis-acting elements at the 5′ and 3′ ends, along with the genes encoding all non-structural proteins controlled by the cytomegalovirus immediate-early promoter (CMVp). These viral RNA subgenomes, driven by the CMVp, are capable of self-replication in transfected cells but do not generate infectious particles [12,13]. Consequently, flavivirus replicons can be utilized as vectors for expressing foreign genes and exploring gene therapy applications.

Various techniques, such as lipid-based transfection, microinjection, electroporation, particle bombardment, and virus-like particles (VLPs), can be employed to deliver RNA- and DNA-launched replicons into cells [14,15,16]. VLPs containing self-replicating replicon-driven RNAs can be used to deliver flavivirus replicons into target cells, where the replicon-driven RNA is released and initiates replication in both in vitro and in vivo systems. In our previous studies [9,10,11], we successfully generated JEV and ZIKV single-round infectious particles (SRIPs) carrying replicon-driven RNAs using reverse genetic techniques. These particles can be engineered as delivery vehicles for flavivirus replicons containing foreign genes in cells. It is important to note that when using ZIKV replicons as vectors for heterogeneous gene expression, reporter genes, safety, and replication efficacy should be carefully considered.

This study aimed to minimize the size of ZIKV replicons by deleting viral genes that are not required for the self-replication of replicon-driven RNAs. The ZIKV Natal RGN replicon (ZIKV rep) that was created by combining various components into the pBR322 plasmid includes several components: the CMVp, ZIKV cDNA fragment 5′-UTR-C-NS1 to NS5-3′UTR, green fluorescent protein (GFP), self-cleaving peptide FMDV-2A (F-2A), hepatitis delta virus ribozyme (HDVr), and bovine growth hormone polyA signal (BGH-pA). This study constructed ZIKV mini-rep 1 containing ZIKV cDNA fragment (5′UTR-C-RdRp-3′UTR), mini-rep 2 containing ZIKV cDNA fragment (5′UTR-C-NS5-3′UTR), and mini-rep 3 containing ZIKV cDNA fragment (5′UTR-C-NS4B-NS5-3′UTR). These DNA-launched mini-replicons were designed as expression vectors to carry the genes of interest, like reporter genes, and then transfected into ZIKV prM-E-expressing cell lines to synthesize mini-replicon driven RNAs encapsidated into prM-E VLPs as ZIKV SRIPs carrying mini-replicon-driven RNAs. Subsequently, the virus yield, infectivity, cell susceptibility, RNA self-replication, and kinetic expression of heterologous genes of ZIKV mini-replicon-based SRIPs were examined by the assays of Western blotting, real-time RT-PCR, TCID50, and fluorescence resonance energy transfer (FRET). In addition, the in vivo expression of heterologous genes by ZIKV mini-replicons was demonstrated using a mouse model via the intravenous injection of ZIKV mini-rep 3-hACE2 SRIPs carrying human *ACE2* gene.

## 2. Materials and Methods

### 2.1. Cells

The cell lines used were TE671 (human, rhabdomyosarcoma/muscle), HEK293T (human embryonic kidney), A549 (human lung carcinoma epithelial), and SF268 (human glioblastoma) cells. The cell lines were cultured in minimum essential medium supplemented with 10% fetal bovine serum, 2 mM glutamine, 1 mM pyruvate, and 1× penicillin-streptomycin at 37 °C with 5% CO_2_, as previously described in our publications [9,10,11]. In particular, the TE671-ZIKV prM-E stable cells were maintained using the media described in our previous publications [10,11] and further transfected with different ZIKV mini-replicons to produce ZIKV mini-replicon SRIPs, respectively.

### 2.2. Construction of ZIKV Mini-Replicons Comprising the Green Fluorescent Protein Gene

**Figure 1:**
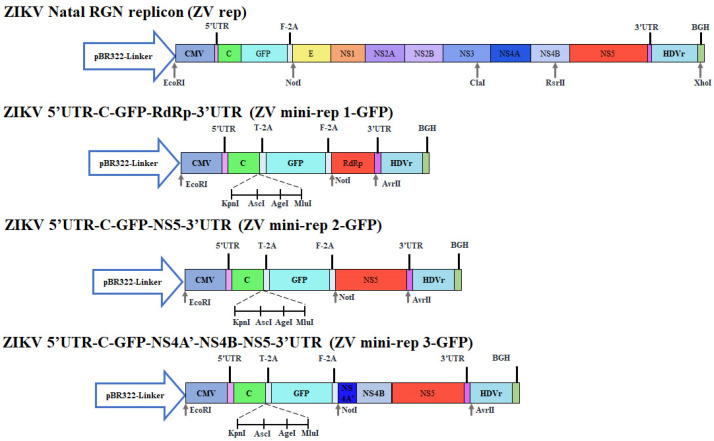
The construction and gel electrophoresis analysis of ZIKV mini-replicons containing the GFP reporter. Fragments F1 and F2 were amplified, digested, ligated, and transformed into *E. coli* cells, resulting in the creation of ZIKV mini-rep 1-GFP. Additional fragments F3 and F4, encompassing NS5 MTase and partial NS4A, NS4B, and NS5 MTase domain, were also amplified, digested, and ligated with ZIKV mini-rep 1-GFP, leading to the generation of ZIKV mini-rep 2-GFP and ZIKV mini-rep 3-GFP clones.

The ZIKV Natal RGN replicon (ZIKV rep, as shown in Figure 1) consisted of several components cloned into the pBR322 plasmin, including CMVp, cDNA fragments of the ZIKV Natal RGN genome (GenBank accession number KU527068) with the prM-E genes removed, GFP, F-2A, HDVr, and BGH-pA. The construction process of ZIKV Natal RGN replicon was previously described in our publication [10,11]. To create the ZIKV mini-replicon 1-GFP (ZIKV mini-rep 1-GFP, as shown in Figure 1), Fragment 1 (F1) containing RdRp-3′UTR-HDVr-BGH-pA-pBR322-5′UTR-C was firstly amplified using PCR with DreamTaq Green PCR Master Mix, the ZIKV Natal RGN replicon template, the forward primer NotI_Rsr_ZIKV nt7830-For, and the reverse primer ZIKV C_Kpn1_Asc1-Rev (Supplemental Table S1). Next, Fragment 2 (F2) containing the Thosea asigna virus 2A-like peptide (T2A), GFP, and Foot-and-Mouth Disease virus (FMDV) 2A peptide was amplified using PCR with the Asc1_T2A_GFP-For forward primer and the F2A_Not1-Rev reverse primer (Supplemental Table S1). Both F1 and F2 contained AscI and NotI restriction enzyme sites at the 5′ and 3′ ends, respectively. After digesting F1 and F2 with AscI and NotI, the fragments in a 1:3 or 1:5 ratio were mixed and ligated using T4 DNA Ligase reaction overnight at 16 °C (Thermo Scientific, Waltham, MA, USA). A total of 1 μL of the ligation reaction was then transformed into *E. coli* 10B competent cells and selected on LB agar plates containing ampicillin at 37 °C for 16 to 18 h. After checking the size of the clones via gel electrophoresis, the recombinant clones of ZIKV mini-rep 1-GFP were sequenced to confirm successful construction and stored at −70 °C. To construct the ZIKV mini-replicon 2-GFP (ZIKV mini-rep 2-GFP, as shown in Figure 1), Fragment 3 (F3), which contained the MTase of NS5, was amplified by PCR with the primer pair ZIKV NS5 MTase_Not1-For and the reverse primer ZIKV NS5(8406) AvrII-Rev (Supplemental Table S1). The resulting fragments of ZIKV mini-rep1-GFP and F3 that were digested using Not1 and AvrII were ligated using a T4 DNA Ligase reaction overnight at 16 °C. The ligation reaction was then transformed into *E. coli* 10B competent cells, and the transformed cells were selected on LB agar plates containing ampicillin overnight. To confirm the successful construction of the ZIKV mini-rep 2-GFP clones, the clones’ sizes were determined by gel electrophoresis, and they were subjected to sequencing analysis to validate the construction. A similar approach was used to construct the ZIKV mini-rep 3-GFP (Figure 1); Fragment 4 (F4), which contained C-terminal NS4A, NS4B, and MTase NS5, was amplified by PCR with the primer pair NS4A2′_NotI For and the reverse primer ZIKV NS5(8406) AvrII-Rev (Supplemental Table S1) and then cloned into ZIKV mini-rep1-GFP. After transformation, antibiotic selection, and sequencing analysis, the recombinant clones of ZIKV mini-rep 3-GFP were successfully constructed and then stored at −70 °C.

### 2.3. Construction of ZIKV Mini-Replicons Comprising the Cyan Fluorescent Protein-Linker- Yellow Fluorescent Protein (CFP/YFP, CYP) Fusion Gene or Human ACE2 Gene

To generate mini-rep 2-CYP and mini-rep 3-CYP (Supplemental Figure S1), ZIKV mini-rep 1-no reporter was first constructed. This was achieved by performing PCR using the mini-rep 1-GFP template and the primer pairs Cla1_EcoRV_3′GFP-For and 5′GFP_Mlu1_Cla1-Rev (Supplemental Table S1). The PCR product, also called Fragment F5, was then subjected to digestion by ClaI enzyme. Subsequently, the resulting fragments were self-ligated using T4 DNA Ligase, and a clone of ZIKV mini-rep 1-no reporter was selected from this ligation reaction. Next, the CFP/YFP gene fragment (F6) was amplified through PCR using a previously reported template [17] and the primer pairs KpnI_CFP-For and YFP_AscI-Rev (Supplemental Table S1). This fragment was then inserted into ZIKV mini-rep 1-no reporter, resulting in a clone named ZIKV mini-rep 1-CYP. Subsequently, the F3 and F4 fragments were individually cloned into the Not1/AvrII sites of ZIKV mini-rep 1-CYP. These clones were designated as mini-rep 2-CYP and mini-rep 3-CYP, respectively. Sequencing analysis was performed to confirm the successful construction of the recombinant clones of ZIKV mini-replicons containing the CYP gene, and confirmed clones were stored at −70 °C for further use. In addition, ZIKV mini-replicons containing the human *ACE2* gene were created. The human *ACE2* gene was amplified by PCR using a reported template [17] and the primer pairs KpnI-ACE2-For and ACE2 AscI-Rev (Supplemental Table S1). The PCR products or the human *ACE2* (hACE2) gene were then inserted into the KpnI/AscI sites of ZIKV mini-rep 2-GFP and ZIKV mini-rep 3-GFP, resulting in mini-replicons named ZIKV mini-rep 2-hACE2 and ZIKV mini-rep 3-hACE2, respectively. Sequencing analysis was performed to confirm the successful construction of the recombinant clones of ZIKV mini-replicons containing the CYP or hACE2 gene, and confirmed clones were stored at −70 °C for further use.

### 2.4. Production of ZIKV Mini-Replicon-Driven RNA-Based SRIPs Expressing GFP, CYP, or hACE2

TE671-ZIKV prM-E cell line, which co-expresses ZIKV prM and E proteins, was established in our previous study [10]. TE671-ZIKV prM-E cells were grown in 6-well plates until 90% confluence and then transfected with mini-replicons of ZIKV that contained either GFP, CYP, or hACE2 genes using Lipofectamine LTX. The expression of mini-replicon-driven proteins, including GFP, CYP, hACE2, and ZIKV NS5 proteins, was examined by immunofluorescence microscopy and immunofluorescence assay using primary antibodies against ZIKV NS5 and human ACE2 (GeneTex, Hsinchu, Taiwan) and secondary AF546 goat anti-rabbit IgG antibodies (from Thermo Fisher Scientific). The ZIKV prME/mini-rep SRIPs (ZIKV mini-rep P0 SRIPs) were collected from the media of the transfected TE671-ZIKV prM-E cells 72 h post-incubation. Passage 1 (P1) of the ZIKV prME/mini-rep SRIPs (ZIKV mini-rep P1 SRIPs) was collected from the media of TE671-ZIKV prM-E cells infected with the ZIKV mini-rep P0 SRIPs, condensed using the PEG Virus Precipitation Kit (Sigma-Aldrich, St. Louis, MO, USA), and stored at −80 °C. 

To confirm the presence of mini-replicon-driven self-replicating RNA genomes within the ZIKV mini-rep P1 SRIPs, real-time RT-PCR assays were conducted using specific primer pairs for ZIKV NS5 and human *ACE2* (listed in Supplementary Table S2). Additionally, the E, prM/M, and C proteins present on the surface of the ZIKV mini-rep P1 SRIPs were analyzed using Western and dot blotting assays with specific antibodies against E, prM/M, and C proteins, as described in our previous studies [10,18]. Furthermore, the infectious titers of the P1 SRIP stock were determined through a median tissue culture infectious dose (TCID50) assay. Serial dilutions of the P1 SRIP stock were added to 96-well plates containing a 90% confluent monolayer of TE671-ZIKV prM-E cells. Following incubation for 72 h at 37 °C, any cytopathic effects (CPE) in each well were observed and recorded to determine the TCID50 titer of the P1 SRIP stock.

### 2.5. Transient Reporter Expression Kinetics and Cytopathic Ability by ZIKV Mini-rep 2-CYP and 3-CYP SRIPs 

The CYP reporter expression driven by ZIKV mini-rep 3-CYP SRIPs was evaluated in HEK293T, TE671, A549, and SF268 cells at MOIs of 0.1, 1, and 2. To quantify the FRET signal of CYP fluorescence, cell lysates were prepared from the infected cells and transferred to a 96-well black plate on Days 1, 2, and 3 post-infection to examine the expression of the CYP reporter, which is a fusion protein of CFP and YFP. The FRET signal of CYP was measured using a SpectraMax Multi-Mode Microplate Reader (Molecular Devices San Jose, CA, USA) with excitation and emission wavelengths set at 436–528 nm. Additionally, the viability of cells infected with ZIKV mini-rep 3-CYP SRIPs at different MOIs was determined on Day 3 post-infection using the MTT (3-(4,5-dimethyl-2-thiazolyl)-2,5-diphenyl-2-H-tetrazolium bromide) assay (Sigma-Aldrich). The absorbance of the mock-infected cells was used as a control to represent 100% cell viability, as described in our previous study [18].

### 2.6. In Vivo Expression of hACE2 in the Mice with an Intravenous Injection of ZIKV prME/Mini-rep 3-hACE2 SRIPs

A study was conducted at China Medical University using a mouse model to investigate the effects of an intravenous injection of ZIKV prME/mini-rep 3-hACE2 SRIPs. The Institutional Animal Care and Use Committee (IACUC) approved the study protocol on June 9th, 2020 (Animal Use Protocol No. CMUIACUC-2020-293). Female BALB/c mice, aged 6 weeks, were divided into two groups. Group I received a 50 µL intravenous injection of PBS on Day 1. Group II received a 50 µL intravenous injection of ZIKV prME/mini-rep 3-hACE2 SRIPs (1.5 × 10^6^ TCID50/mL) on Day 1. On Day 6, the mice were euthanized, and their lungs and brains were collected for further analysis. The collected tissues were fixed in 10% neutral buffered formalin overnight. Subsequently, they underwent a series of steps for histopathology assays. The tissues were dehydrated using different concentrations of ethanol and then embedded in paraffin. Thin sections (ranging from 4 to 15 µm) were obtained from the paraffin-embedded tissues using a microtome. These sections were deparaffinized, rehydrated, and stained with SPECTRA H&E Stains to visualize tissue morphology. The staining process was performed using the BOND-III Fully Automated IHC and ISH Stainer by Leica. After staining, the sections were dehydrated, cleared, air-dried, and mounted. For the immunohistochemistry assay, the sections underwent deparaffinization, rehydration, and heat-induced antigen retrieval using SPECTRA H&E Stains by Leica on the BOND-III Fully Automated IHC and ISH Stainer. Primary antibodies against hACE2 were applied to the sections and incubated overnight. To block endogenous peroxidase activity, the sections were treated with 3% H_2_O_2_. Biotinylated universal antibodies from the Bond Enzyme Pretreatment Kit (Leica, Wetzlar, Germany) were added, followed by treatment with the Bond Enzyme Pretreatment Kit reagent. The presence of immune complexes in the sections was visualized by the development of a dark brown-black precipitate using the Bond Enzyme Pretreatment Kit. Finally, the sections were counterstained with hematoxylin and mounted. High-resolution digital images of the tissue sections were captured using the Pathology Scanner Second Generation SG300 by Philips. These images were then analyzed using the PMA.start software provided by Pathomation for whole-slide imaging analysis.

### 2.7. Statistical Analysis

The data presented in this study were obtained from three independent experiments. To analyze the data, a one-way analysis of variance (ANOVA) was performed, followed by Scheffe’s post-hoc test. Statistical significance was determined by considering a *p*-value of less than 0.05.

## 3. Results

### 3.1. Construction of ZIKV Mini-Replicons Encoding the Gene of Interest

ZIKV mini-replicon-based vectors for heterogeneous gene expression were generated using the ZIKV Natal RGN replicon (ZIKV rep) described in our previous publication [10] as a starting point (Figure 1). The construction of the ZIKV mini-rep 1-GFP involved removing specific genes (NS1, NS2A, NS2B, NS3, NS4A, NS4B, and MTase) and retaining cis-acting essential regions (5′-UTR, C, RdRp, and 3′-UTR) for RNA self-replication. In addition, a multiple cloning site (MCS) and T-2A peptide sequence were introduced for cloning and expressing heterogeneous genes. The resulting mini-replicon was constructed by ligating PCR products F1 and F2 (Supplemental Figure S2, lanes 4 and 6). Recombinant ZIKV mini-rep 1-GFP clones were confirmed for size (8.8 kb) through gel electrophoresis and validated by sequencing (Supplemental Figure S1A). The successful clones contained various components in the following order: CMVp, 5′-UTR, C, MCS, T-2A, GFP, F-2A, RdRp, 3′-UTR, HDVr, and BGH-pA (Figure 1). To assess the impact of NS4B and MTase on the efficiency of self-replication in mini-replicon-driven RNAs, the MTase gene and NS4A’-NS4B-MTase genes were separately cloned into the NotI/AvrII sites of ZIKV mini-rep 1-GFP. Gel electrophoresis and sequencing confirmed the presence of recombinant clones for ZIKV mini-rep 2-GFP and ZIKV mini-rep 3-GFP (Supplemental Figure S2, lanes 9–12).

### 3.2. The Involvement of ZIKV NS4B and MTase in RdRp-Mediated Replication of Mini-Replicon-Driven RNAs

**Figure 2:**
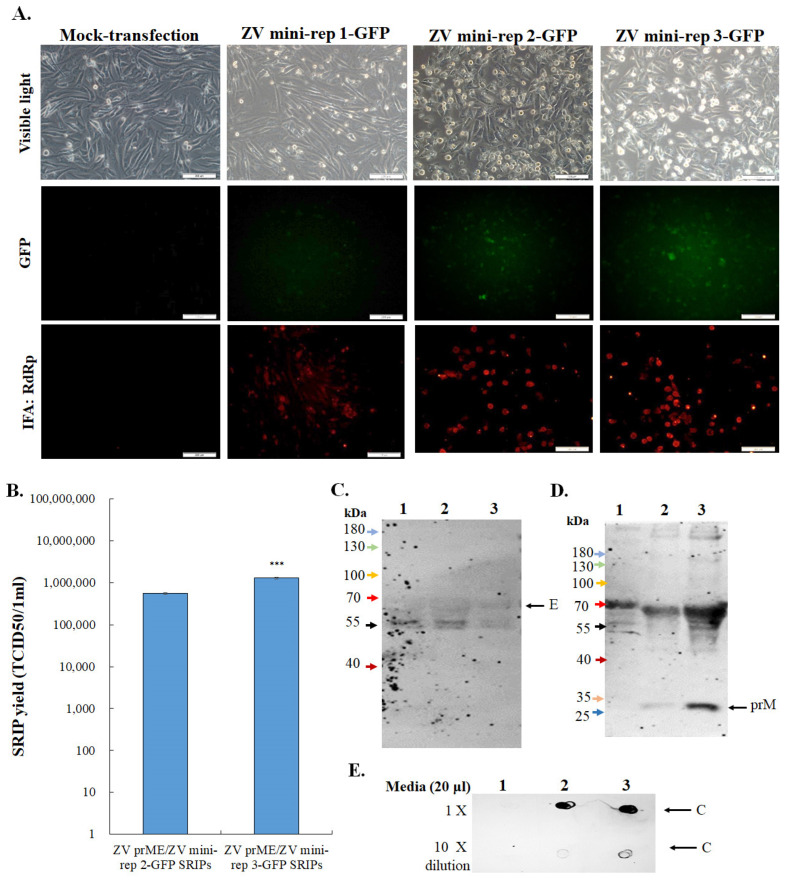
The quantitative and qualitative analysis of ZIKV mini-replicon-based SRIPs containing the GFP reporter. Cytopathic effect, GFP expression, and ZIKV RdRp expression in ZIKV prM and E co-expressing cells transfected with indicated ZIKV mini-replicons were assessed using fluorescence microscopy and immunofluorescent staining with anti-ZIKV RdRp antibodies, followed by labeling with Alexa Fluor 546-conjugated secondary antibodies (**A**). The titer and protein composition of ZIKV mini-rep 2-GFP and 3-GFP SRIPs obtained from the supernatant of packaging cells transfected with ZIKV mini-rep 2-GFP, and 3-GFP were analyzed using TCID50 assay (**B**), Western blotting with anti-ZIKV-E and prM antibodies (**C**,**D**), and dot blot with anti-ZIKV-C antibodies (**E**). Lane 1, control media; Lane 2, mini-rep 2-GFP SRIP; Lane 3, mini-rep 3-GFP SRIP. ***, *p*-value < 0.001. Scale bar, 200 μm.

To investigate the self-replication of Zika virus (ZIKV) mini-replicon-driven RNAs, we utilized TE671-ZIKV prM-E cells, which were previously established in our other study [10], to express ZIKV prM and E proteins. These cells were transfected with ZIKV mini-replicons individually, and subsequent analysis was conducted to examine the cytopathic effect, as well as the expression of green fluorescent protein (GFP) and ZIKV RdRp protein in the transfected cells (Figure 2A). After 72 h of transfection, noticeable cytopathic changes and robust green fluorescence spots were observed in the TE671-ZIKV prM-E cells transfected with ZIKV mini-rep 2-GFP and ZIKV mini-rep 3-GFP, respectively (Figure 2A). In contrast, the cells transfected with ZIKV mini-rep 1-GFP showed only slight levels of cytopathic effect and green fluorescence spots compared to the mock-transfected cells. Immunofluorescent staining analysis revealed similar patterns of ZIKV RdRp protein expression in the cells transfected with the above-mentioned mini-replicons. (Figure 2A). To evaluate the self-replication of mini-replicon-driven RNAs, passage 0 (P0) of the ZIKV mini-replicon-based self-replicating infectious particles (ZIKV mini-rep P0 SRIPs) was collected from the supernatant of the mini-replicon-transfected prM-E co-expressing cells 72 h post-transfection. The prM-E co-expressing cells were then infected with these P0 SRIPs to generate passage 1 (P1) of the ZIKV mini-replicon-based SRIPs (ZIKV mini-rep P1 SRIPs). Subsequently, the yield titer of ZIKV mini-rep 2-GFP and 3-GFP P1 SRIPs was determined using a TCID50 assay with prM-E co-expressing cells. The titer was found to be 5.5 × 10^5^ TCID50/mL for ZIKV mini-rep 2-GFP P1 SRIPs and 1.3 × 10^6^ TCID50/mL for ZIKV mini-rep 3-GFP P1 SRIPs (Figure 2B). Additionally, Western blotting and dot-blotting assays confirmed the presence of the structural proteins E, (pr)M, and C in the presence of ZIKV mini-rep 2-GFP and 3-GFP P1 SRIPs (Figure 2C–E).

### 3.3. Engineering ZIKV Mini-Replicon Vectors

**Figure 3:**
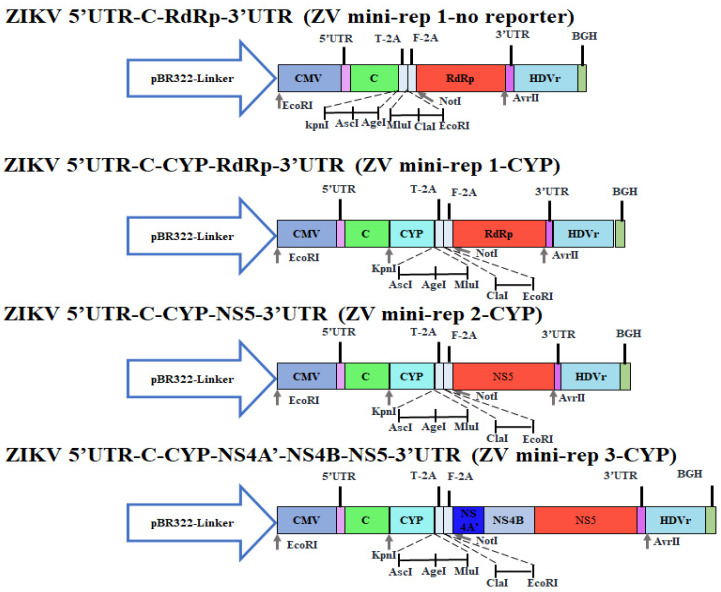
Construction of ZIKV mini-replicons containing the CYP reporter (cyan fluorescent protein-linker-yellow fluorescent protein). ZIKV mini-rep 1-no reporter was created by self-ligating the PCR-amplified fragment F5 using the mini-rep 1-GFP template and specific primers. Subsequently, a CFP/YFP gene fragment (F6) was amplified and inserted into ZIKV mini-rep 1-no reporter, resulting in ZIKV mini-rep 1-CYP. The F3 and F4 fragments were individually cloned into specific sites of ZIKV mini-rep 1-CYP, generating ZIKV mini-rep 2-CYP and ZIKV mini-rep 3-CYP, respectively.

**Figure 4:**
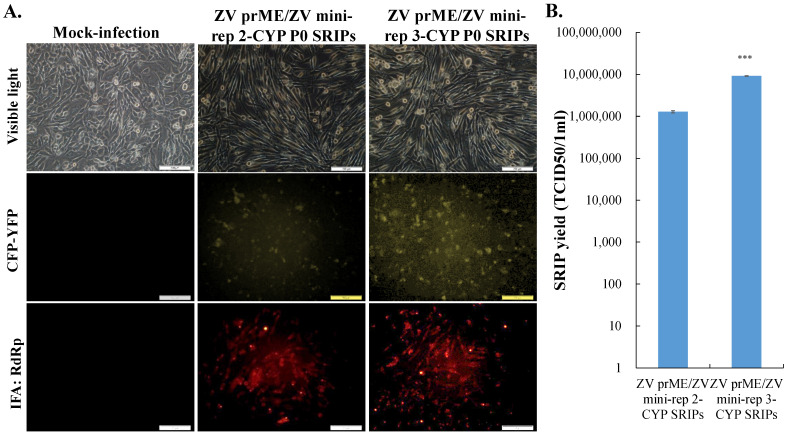
The analysis of infectivity and quantification of ZIKV mini-rep 2-CYP and 3-CYP SRIPs. The cytopathic effect, CYP expression, and ZIKV RdRp expression in ZIKV prM and E co-expressing cells infected with ZIKV mini-rep 2-CYP and 3-CYP SRIPs were analyzed using fluorescence microscopy and immunofluorescent staining (**A**). The titer of ZIKV mini-rep 2-CYP and 3-CYP SRIPs was determined by assessing the supernatant of packaging cells infected with ZIKV mini-rep 2-CYP and 3-CYP SRIPs using the TCID50 assay (**B**). ***, *p*-value < 0.001. Scale bar, 200 μm.

To create the multiple-cloning sites and clone the other reporter gene in ZIKV mini-replicon vectors, ZIKV mini-rep 1-no reporter and ZIKV mini-rep 1-CYP were constructed by ligating F5 (ZIKV mini-rep 1-GFP with the GFP gene removed) alone or with F6 (CFP/YFP fusion gene) (Figure 3). The resulting recombinant clones were confirmed for size and sequenced (Supplemental Figure S1B). Fragments F3 and F4 were subsequently inserted into the NotI/AvrII sites of the ZIKV mini-rep 1-CYP vector, leading to the development of ZIKV mini-rep 2-CYP and ZIKV mini-rep 3-CYP. Their sizes were verified, and sequencing confirmed their construction (Supplemental Figure S3). Overall, the process involved amplification, digestion, ligation, transformation, and confirmation through size analysis and sequencing to create the ZIKV mini-replicons. A similar process was followed to generate the P0 of ZIKV mini-rep 2-CYP and 3-CYP SRIPs from prM-E co-expressing cells transfected with mini-rep 2-CYP and 3-CYP, respectively. These P0 SRIPs were then used to infect prM-E co-expressing cells, generating P1 SRIPs (Figure 4). Similar to ZIKV mini-rep 3-GFP P0 SRIPs, ZIKV mini-rep 3-CYP P0 SRIPs induced higher levels of cytopathic effect, CFP-YFP, and ZIKV RdRp expression in infected cells, resulting in a superior yield of P1 SRIPs compared to ZIKV mini-rep 2-CYP P0 SRIPs. The findings exhibited the ability of ZIKV mini-rep 3-GFP and 3-CYP RNAs, under the control of NS4B, MTase, and RdRp, to replicate themselves autonomously. This led to a significant generation of ZIKV mini-rep 3-GFP and 3-CYP SRIPs.

### 3.4. Comparative Analysis of Heterologous Gene Expression in Infected Cells with ZIKV SRIPs Carrying Mini-Replicon-Driven RNAs 

**Figure 5:**
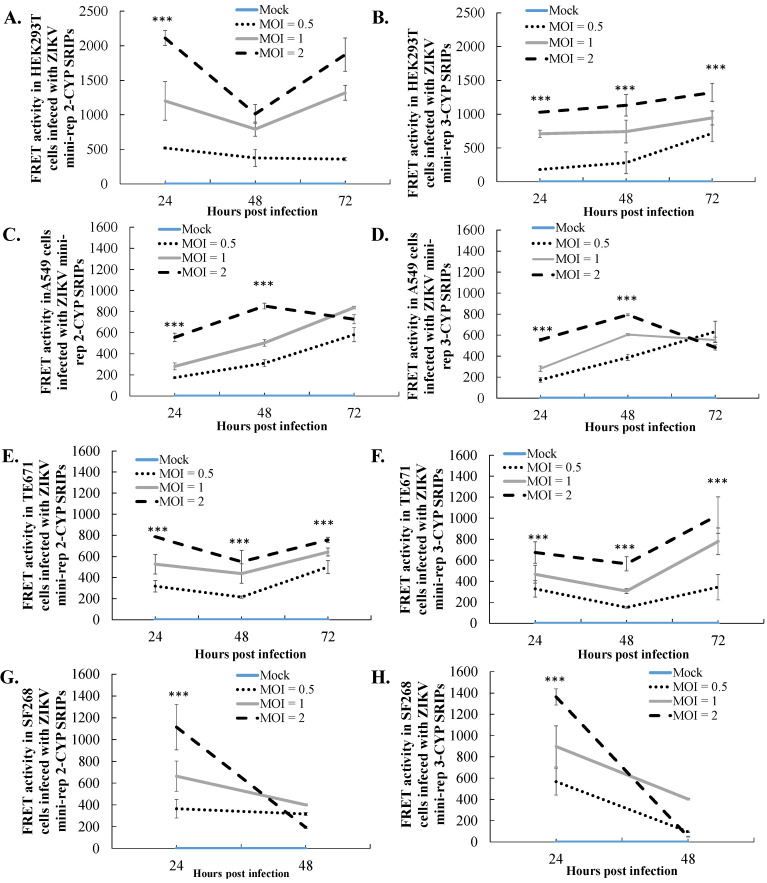
CYP expression kinetics in the cells infected with ZIKV mini-rep 2-CYP and 3-CYP SRIPs at different MOIs. The evaluation was performed in four types of cell lines: HEK293T (**A**,**B**), A549 (**C**,**D**), TE671 (**E**,**F**), and SF268 (**G**,**H**) cells. The MOIs tested were 0.1, 1, and 2. To assess the FRET signal of CYP fluorescence, cell lysates were prepared from the infected cells and transferred to a 96-well black plate on Days 1, 2, and 3 post-infection. The FRET signal of CYP was measured using a SpectraMax Multi-Mode Microplate Reader with excitation and emission wavelengths set at 436–528 nm. ***, *p*-value < 0.001.

**Figure 6:**
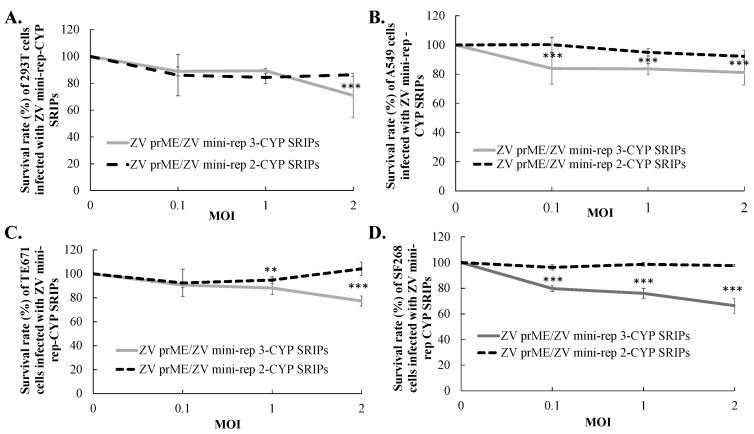
The survival rates of cells infected with ZIKV mini-rep 2-CYP and 3-CYP SRIPs were evaluated at different MOIs in four types of cell lines: HEK293T (**A**), A549 (**B**), TE671 (**C**), and SF268 (**D**) cells. The tested MOIs included 0.1, 1, and 2. To assess the survival rates of infected cells, the MTT assay was performed on Day 2 for SF268 cells and on Day 3 for HEK293T, A549, and TE671 cells after infection. **, *p*-value < 0.01. ***, *p*-value < 0.001.

To investigate the expression kinetics of reporter genes driven by self-replicating RNAs of mini-replicons 2 and 3, we conducted experiments using HEK239T, TE671, A549, and SF268 cells. These cells were infected with ZIKV mini-rep 2-CYP and 3-CYP SRIPs at MOIs of 0.1, 1, and 2. The detection of FRET signals allowed us to assess the expression levels of the reporter gene CYP. We observed that the peak of the CYP signals driven by mini-replicons 2 and 3 was found in HEK239T and TE671 cells 72 h post-infection with ZIKV mini-rep SRIPs (Figure 5A,B,E,F). In A549 cells, the infection with ZIKV mini-rep SRIPs at MOIs of 1 and 2, but not at an MOI of 0.1, led to a decrease in the CYP signals driven by mini-replicon 3 at 72 h post-infection (Figure 5C,D). Furthermore, in SF268 cells, lower CYP signals driven by mini-replicons 2 and 3 were observed 48 h post-infection compared to 24 h post-infection with ZIKV mini-rep SRIPs at MOIs of 0.1, 1, and 2 (Figure 5G,H). A survival analysis of the infected cells revealed that the infection with ZIKV mini-rep 2 and 3 SRIPs resulted in noticeable cytotoxic effects in TE671, A549, and SF268 cells (Figure 6). The cells infected with ZIKV mini-rep 2 SRIPs exhibited a higher survival rate compared to cells infected with ZIKV mini-rep 3 SRIPs at the same MOI (Figure 6). In addition, when cells were infected with ZIKV mini-rep 3 SRIPs, the survival rate of infected cells showed a dependence on the MOI. Notably, at an MOI of 2, ZIKV mini-rep 3 SRIPs resulted in a reduction of over 30% in the cell survival rate of SF268 cells (Figure 6D). The experiments involving the infection of cells with ZIKV mini-replicon 2-CYP and 3-CYP SRIPs provided valuable insights into the expression kinetics of the reporter gene CYP and the cytotoxic effects induced by mini-replicons 2 and 3 in various cell types. The results shed light on the dynamic patterns of CYP expression and the potential harm caused by these mini-replicons when introduced into the cells by ZIKV mini-rep SRIPs.

### 3.5. In Vivo Expression of hACE2 Delivered by ZIKV Mini-rep 3 SRIPs 

**Figure 7:**
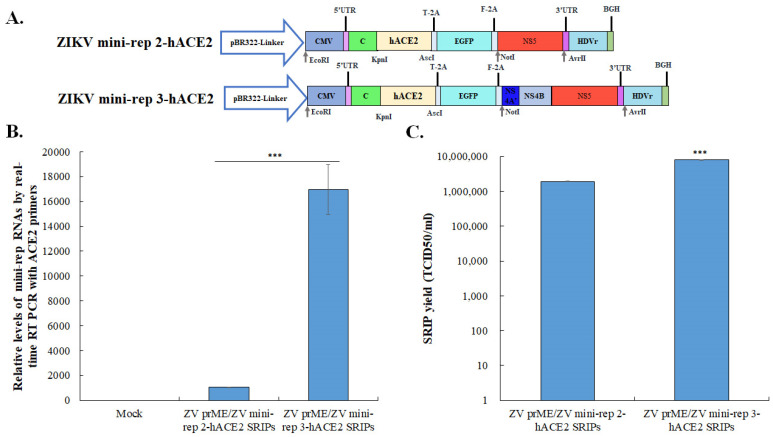
The assessment of infectivity and quantification of ZIKV mini-rep 2-hACE2 and 3-hACE2 SRIPs. The hACE2 gene was amplified using PCR and inserted into the KpnI/AscI sites of ZV mini-rep 2-GFP and ZV mini-rep 3-GFP, resulting in the creation of mini-replicons named ZV mini-rep 2-hACE2 and ZV mini-rep 3-hACE2, respectively (**A**). The mRNA expression levels of hACE2 in cells infected with ZIKV mini-rep 2-hACE2 and 3-hACE2 P0 SRIPs were detected using real-time RT-PCR (**B**). Viral titers of ZIKV mini-rep 2-hACE2 and 3-hACE2 P1 SRIPs were determined using TCID50 assays (**C**). ***, *p*-value < 0.001.

**Figure 8:**
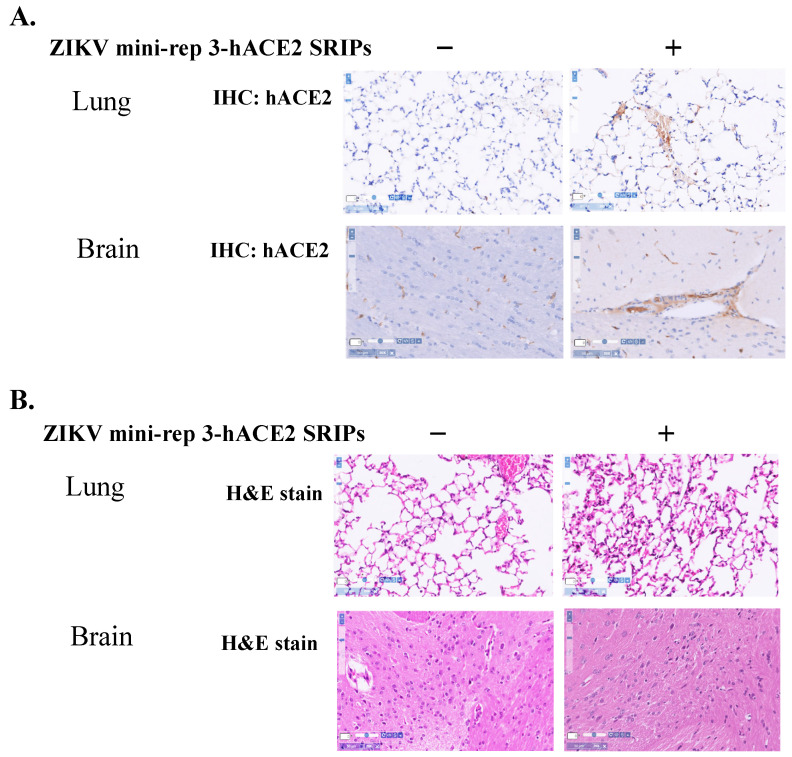
Immunohistochemistry analysis and histopathology examination of the lung and brain tissues obtained from two groups of mice. The groups included Group I (PBS injections), and Group II (ZV prME/mini-rep 3-hACE2 SRIPs. Immunohistochemistry was performed with primary antibodies against hACE2 after deparaffinization and rehydration of lung tissue sections (**A**). Tissue morphology was visualized through staining with SPECTRA H&E Stains (**B**). Scale bar, 50 μm.

To investigate the expression of foreign genes delivered by ZIKV mini-replicon SRIPs, a ZIKV mini-rep 2-hACE2- and 3-hACE2 SRIP-based approach was employed (see Figure 7 and Figure 8). The initial step involved generating the P0 of the ZIKV mini-rep 2-hACE2 and 3-hACE2 SRIPs by transfecting mini-replicons 2-hACE2 and 3-hACE2 into the cells expressing ZIKV prM-E, respectively. The P0 hACE2 SRIP infection caused mild cytopathic effects in prM-E co-expressing cells, in which mini-rep 3-hACE2 SRIP infection showed high levels of hACE2 mRNA expression (Figure 7B), resulting in a higher yield of P1 mini-rep 3-hACE2 SRIPs compared to P1 mini-rep 2-hACE2 SRIPs. To further evaluate in vivo hACE2 expression, an assay was conducted using BALB/c mice (Figure 8). The mice were divided into two groups: Group I received intravenous injections of PBS on Day 1 as the control group, while Group II received intravenous administration of ZIKV prME/mini-rep 3-hACE2 SRIPs on Day 1. On Day 6, comprehensive analyses were performed on lung and brain samples collected from each group. IHC staining using hACE2-specific antibodies (Figure 8A) was carried out on lung and brain tissues from different groups to examine the expression of hACE2 mediated by ZIKV mini-rep 3-hACE2 SRIPs. The IHC results revealed that hACE2 expression was detected in the perivascular and alveolar regions of lung tissues in mice from Group II but not Group I. Additionally, the presence of the hACE2 protein was exclusively observed in the brain capillaries of mice from Group II. A histological examination using H&E staining was performed on mouse lung and brain tissues to further investigate the impact of ZIKV mini-rep 3-hACE2 SRIPs on pulmonary and brain inflammation (Figure 8B). Group II mice, which received intravenous injections of ZIKV mini-rep 3-hACE2 SRIPs, exhibited mild inflammation in the lungs and brain. In contrast, no significant alterations were observed in the lung tissues of Group I mice injected with PBS intravenously. These findings demonstrate that the intravenous injection of ZIKV mini-rep 3-hACE2 SRIPs in mice resulted in hACE2 expression in the lungs and brain. Thus, it suggests that ZIKV mini-replicon SRIPs have the potential to serve as in vivo gene delivery vehicles.

## 4. Discussion

This study represents the first investigation into the use of ZIKV mini-replicons as viral vectors for in vitro and in vivo expressing various genes, including reporters (such as GFP and CFP) and hACE2 (Figure 1, Figure 2, Figure 3, Figure 4, Figure 5, Figure 6, Figure 7 and Figure 8). The study demonstrated the successful construction of ZIKV mini-replicons and their application in ZIKV prM-E co-expressing cells to generate ZIKV mini-replicon based SRIPs. Transfection of these mini-replicons or infection with ZIKV mini-rep SRIPs revealed the crucial involvement of ZIKV MTase domain of NS5 and NS4B in facilitating efficient RdRp-mediated self-replication of the ZIKV mini-replicon-driven RNAs, leading to a significant expression of the heterogeneous genes. The physical linkage between the MTase and RdRp domains within NS5 confers specificity for viral cap methylation during flavivirus RNA synthesis [19]. The MTase domain not only performs methylations but also plays a significant role in stimulating both the de novo initiation and elongation phases of RNA synthesis [20,21]. Additionally, MTase enhances the affinity of NS5 for the single-stranded RNA template. This study highlighted the involvement of MTase in RdRp-mediated self-replication of Zika virus mini-replicon-driven RNAs, further supporting the intricate interplay between the MTase and RdRp domains of NS5 [20,21]. NS4B, a transmembrane protein, assumes a critical role in establishing the viral replication complex and modifying intracellular membranes to create an ideal environment for efficient viral replication. Additionally, NS4B actively participates in viral RNA replication and maintains a close association with NS5 [22]. The findings in our previous study potentially correlate with the results of this study, demonstrating that MTase and NS4B enhance the self-replication of Zika virus mini-replicon-driven RNA synthesis mediated by RdRp.

This study provides valuable insights into the dynamics of foreign gene expression driven by ZIKV mini-replicon-based RNAs in cells infected with ZIKV mini-replicon-based SRIPs, as depicted in Figure 1, Figure 2, Figure 3, Figure 4, Figure 5, Figure 6, Figure 7 and Figure 8. The results reveal distinct expression patterns of the reporter gene CYP in various infected cell lines, with some patterns being dependent on both MOI and the duration of infection. Notably, the infection had deleterious effects on SF268 and A549 cells but not on HEK293T cells, as shown in Figure 5 and Figure 6. This study also highlights the significant potential of ZIKV mini-replicon-based SRIPs as highly efficient gene delivery vehicles capable of targeting a wide range of host cells, based on the analysis of self-replicating RNA levels, virus yield, and cell susceptibility, as illustrated in Figure 2, Figure 4 and Figure 7. The development of replicons from various flaviviruses, such as Kunjin virus (KUNV), Yellow Fever virus (YFV), Dengue virus, Japanese Encephalitis virus (JEV), Tick-borne Encephalitis virus, and West Nile virus, has been previously reported [7,9,23,24]. Some of these replicons have been used as both homologous and heterologous vaccines in animal studies [25,26,27]. For instance, the KUNV replicon system can be delivered as DNA, RNA, or virus-like particles, enabling efficient cell division and the stable expression of foreign genes [8,26]. These findings hold immense promise for various applications of ZIKV mini-replicon-based SRIPs, including enhanced protein production, advanced gene therapy, and the development of novel vaccines. The versatility and effectiveness of these SRIPs make them invaluable tools for advancing research and applications in these fields.

The lack of compatibility between the mouse ortholog of *ACE2* and the SARS-CoV-2 virus poses a challenge for conducting pre-clinical studies in mice. To overcome this hurdle, scientists have employed various strategies, including mouse adaptation of the virus or introducing human *ACE2* (hACE2) expression in mice. Previous studies have utilized transgenic mouse models expressing hACE2 driven by the K18 promoter, as well as mice that received hACE2 delivery through adenovirus (AdV) or adeno-associated virus (AAV) administration to study SARS-CoV-2 infection [28,29,30,31,32]. In this study, we have demonstrated the potential of ZIKV mini-rep 3-hACE2 SRIPs as gene delivery vehicles, in which the intravenous injection of these SRIPs led to hACE2 expression driven by ZIKV mini-replicon 3 in the lungs and brains of the injected mice, as illustrated in Figure 7 and Figure 8. Therefore, this study successfully established a mouse model with ZIKV mini-replicon-mediated hACE2 expression, which can be utilized to investigate SARS-CoV-2 infection. Similar to the AdV-hACE2 and AAV-hACE2 models described in previous studies [28,29,30,31,32], the in vivo hACE2 expression model using ZIKV mini-replicon-based SRIPs offers the advantage of flexibility. It allows for immediate studies in multiple mouse strains without the need for the time-consuming breeding of mice with hACE2 transgenic or knock-in backgrounds. This flexibility facilitates faster research and enables the evaluation of different mouse strains in the context of SARS-CoV-2 infection.

## 5. Conclusions

The findings of this study emphasize the significance of the NS5 MTase domain and NS4B proteins in promoting the effective self-replication of ZIKV mini-replicon-driven RNAs through the RdRp mechanism. Additionally, this study highlights the potential of ZIKV mini-replicons as valuable tools for gene expression in mammalian cells. Furthermore, it showcases the use of ZIKV mini-replicon-based SRIPs as a highly efficient approach for delivering genes, offering promising applications both in vitro and in vivo.

## Data Availability

Not Applicable.

## Supplementary Materials

The following supporting information can be downloaded at: https://www.mdpi.com/article/10.3390/v15081762/s1, Figure S1: The nucleotide sequence analysis of two constructs; Figure S2: Gel electrophoresis analysis of ZIKV mini-replicons containing the GFP reporter; Figure S3: el electrophoresis analysis of ZIKV mini-replicons containing the CYP reporter (cyan fluorescent protein-linker-yellow fluorescent protein); Table S1: Primers for cloning ZIKV mini-replicons; Table S2: Primers for real-time RT PCR assays.
