# Supplementary material for: Development of Zika Virus Mini-Replicon Based Single-Round Infectious Particles as Gene Delivery Vehicles
Source: Viruses. 2023 Aug 18;15(8):1762. doi: 10.3390/v15081762 (PMC10459639; doi:10.3390/v15081762)
Supplement: Supplementary file 1 [file viruses-15-01762-s001.zip › viruses-2543655-supplementary.pdf]

Supplemental materials

Supplemental Table 1. Primers for cloning ZIKV mini-replicons

Primer name	Primer sequences
**ZIKV 5’UTR-C-eGFP-Δ-RdRp-3’UTR (ZV mini-rep 1-GFP)**	
NotI_Rsr_ZV nt7830-For	5’-CGCGGCCGCACGGACCGGTGTCCCGAGGA AGTGCAAAGCTGAGATG-3'
ZV C_Kpn1_Asc1-Rev	5’-GTTGGCGCGCCGGTACCTGCACTCCCACGT CTAGTGAC-3’
Asc1_T2A_GFP-For	5’-CCGGCGCGCCAACCGGTACGCGTGAGGGC AGGGGAAGTCTTCTAACATGCGGGGACGTGG AGGAAAATCCCGGGCCCATGGTGAGCAAGGG CGAGGAGC-3’
F2A_Not1-Rev	5’-CCCTGCGGCCGCGGGCCCGGGGTTGGA-3’
**ZIKV 5’UTR-C-eGFP-Δ-NS5-3’UTR (ZV mini-rep 2-GFP)**	
ZIKV NS5 MTase_Not1-For	5’-ACCGCGGCCGCAGGGGGTGGAACAGGAGA GACCCTG-3’
ZIKV NS5(8406)_AvrII-Rev	5’-TATTTCACTGGCCTCCTAGGCCCGT-3’
**ZIKV 5’UTR-C-eGFP-Δ-NS4A’-NS4B-NS5-3’UTR (ZV mini-rep 3-GFP)**	
NS4A’_NotI For	5’-ATAGCGGCCGCATTGGGCAAGGGCATAGGG-3’
**ZIKV 5’UTR-C-Δ- RdRp-3’UTR (ZV mini-rep 1-no reporter)**	
Cla1_EcoRV_ 3’GFP-For	5’-GTATCGATGATATCACTCTCGGCATGGACG AG-3’
5’GFP_ Mlu1_ Cla1-Rev	5’-TCATCGATACGCGTGAACAGCTCCTCGCCC TT-3’
**ZIKV 5’UTR-C-CFP/YFP-Δ- RdRp-3’UTR (ZV mini-rep 1-CYP)**	
KpnI_CFP-For	5’-ATAGGTACCATGGTTAGCAAGGGCGAG-3’
YFP_AscI-Rev	5’-TATGGCGCGCCGTTTGTACAGTTCGTCC AT-3’
**ZIKV 5’UTR-C-CFP/YFP-Δ-NS5-3’UTR (ZV mini-rep 2-CYP)**	
ZIKV NS5 MTase_Not1-For	5’-ACCGCGGCCGCAGGGGGTGGAACAGGAGA GACCCTG-3’
ZIKV NS5(8406)_AvrII-Rev	5’-TATTTCACTGGCCTCCTAGGCCCGT-3’
**ZIKV 5’UTR-C-CFP/YFP-Δ-NS4A’-NS4B-NS5-3’UTR (ZV mini-rep 3-CYP)**	
NS4A’_NotI For	5’-ATAGCGGCCGCATTGGGCAAGGGCATAGGG-3’
**ZIKV 5’UTR-C-hACE2-eGFP-Δ-NS5-3’UTR (ZV mini-rep 2-hACE2)**	
KpnI-ACE2-For	5’-ATAGGTACCATGTCAAGCTCTTCCTGG-‘3
ACE2 AscI-Rev	5’-ATAGGCGCGCCTTTACCCGGAGACAGGGA GAG-‘3

Supplemental Table 2. Primers for real-time RT PCR assays

Primer name	Primer sequences
**ZIKV NS5**	
ZV-NS5-For	5'-CTTGTGGCTGCTGCGGAGGTCA-3'
ZV-NS5-Rev	5'-GTGGTGGGAGCAAACGGAACTT-3'
**Human ACE2**	
hACE2-For	5'-AGCCCCCCATCGAGAAAACCATC-3'
Hace2-Rev	5'-TGCCCATTGCTCTCCCACTCC-3'

A. ZV mini-rep 1-GFP

ATCAAGCATGGGATATAAAGAGTTCAGAAGATCTGGCTGCCATGCTGAGAATAATCAATGCTAGGAAGGAGAAGAAGA
GACGAGGCGCAGATACTAGTGTCGGAATTGTTGGCCTCCTGCTGACCACAGCTATGGCAGCGGAGGTCACTAGACGTG
Kpn1 AscI AgaI MuI T2A
GGAGTGCAGGTACCGGCGCGCCAACCGGTACGCGTGAGGGCAGGGGAAGTCTTCTAACATGCGGGGACGTGGAGGAA
T2A
AATCCCGGGCCCATGGTGAGCAAGGGCGAGGAGCTGTTCACCGGGGTGGTGCCCATCCTGGTCGAGCTGGACGGCGA
CGTAAACGGCCACAAGTTCAGCGTGTCCGGCGAGGGCGAGGGCGATGCCACCTACGGCAAGCTGACCCTGAAGTTCA
TCTGCACCACCGGCAAGCTGCCCGTGCCCTGGCCCACCCTCGTGACCACCCTGACCTACGGCGTGCAGTGCTTCAGCC
GCTACCCCGACCACATGAAGCAGCACGACTTCTTCAAGTCCGCCATGCCCGAAGGCTACGTCCAGGAGCGCACCATCT
TCTTCAAGGACGACGGCAACTACAAGACCCGCGCCGAGGTGAAGTTCGAGGGCGACACCCTGGTGAACCGCATCGAG
CTGAAGGGCATCGACTTCAAGGAGGACGGCAACATCCTGGGGCACAAGCTGGGGTACAACTACAACAGCCACAACGT
CTATATCATGGCCGACAAGCAGAAGAACGGCATCAAGGTGAACTTCAAGATCCGCCACAACATCGAGGACGGCAGCGT
GCAGCTCGCCGACCACTACCAGCAGAACACCCCCATCGGCGACGGCCCCGTGCTGCTGCCCGACAACCACTACCTGA
GCACCCAGTCCGCCCTGTGCATAGACCCCAATGAGAAGCGCGATCACATGGTCCTGCTGGAGTTCGTGACCGCCGCCG
F2A
GGATCACTCTCGGCATGGACGAGCTGTACAAGAACTTTGATTTGCTCAAGTTGGCAGGAGACGTTGAGTCCAACCCCG
F2A NotI
GGCCCGCGGCCGCATCCCGAGGAAGTGCAAAGCTGAGATGGTTGGTGGGAGCGGGGGATACCTGCAGCCCTATGGGA
AAGGGTCA

B. ZV mini-rep 1-no reporter

CATGGGTATATCAAGATGTTCAAGAAGATCTGGCTGCCATGCTGAGAATAATCAATGCTAGGAAGGAGAAGAA
GAGACGAGGCGCAGATACTAGTGTCGGAATTGTTGGCCTCCTGCTGACCACAGCTATGGCAGCGGAGGTCAC
Kpn1 AscI AgaI T2A
TAGACGTGGGAGTGCAGGTACCGGCGCGCCAACCGGTACGCGTGAGGGCAGGGGAAGTCTTCTAACATGCG
T2A MuI ClaI EcoRV
GGGACGTGGAGGAAAATCCCGGGCCCATGGTGAGCAAGGGCGAGGAGCTGTTCACGCGTATCGATGATATCA
F2A
CTCTCGGCATGGACGAGCTGTACAAGAACTTTGATTTGCTCAAGTTGGCAGGAGACGTTGAGTCCAACCCCG
F2A NotI
GGCCCGCGGCCGCATCCCGAGGAAGTGCAAAGCTGAGATGGTTGGTGGAGCGGGGATACCTGCAGCCCTATG
GAAAGGTCATTGATCTTGGATGTGGCAGAGGGGGCTGGAGTTACTACGCCGCCACCATCCGCAAAGTTCAAG
AAGTGAAAGGATACACAAAAGGAGGCCCTGGTCATGAAGAACCCGTGTTGGTGCAAAGCTATGGGTGGAAC
ATAGTCCGTCTTAAGAGTGGGGTGGACGTCTTTCATATGGCGGCTGAGCCGTGTGACACGTTGCTGTGTGACA
TAGGTGAGTCATCATCTAGTCCTGAAGTGGAAGAAGCACGGACGCTCAGAGTCCTCTCCATGGTGGGGGATT
GGCTTGAAAAAAGACCAGGAGCCTTTTGTATAAAAGTGTTGTGCCCATACACCAGCACTATGATGGAAACCCT
GGAGCGACTGCAGCGTAGTATGGGGGAGGACTGGTCAGAG

Supplemental Figure 1. The nucleotide sequence analysis of two constructs: ZV mini-rep 1-GFP (A) and ZV mini-rep 1-no reporter (B). The figure highlights the presence of restriction enzyme sites and includes the sequences of T2A and F2A peptides, which have been marked for reference.

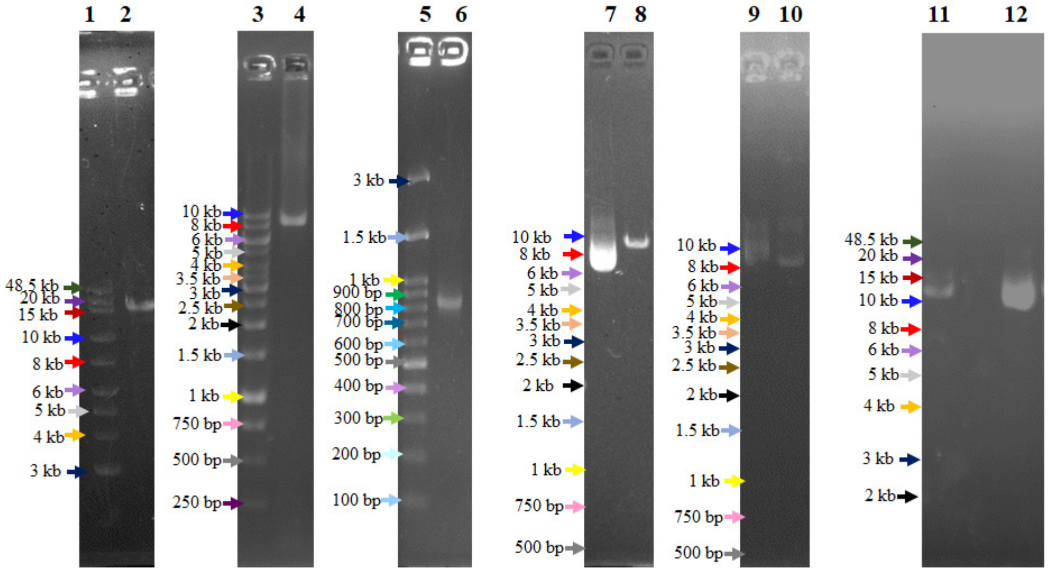

Supplemental Figure 2. Gel electrophoresis analysis of ZIKV mini-replicons containing the GFP reporter. The sizes of the ZIKV Natal RGN replicon, mini-replicons, and cDNA fragments F1 to F4 were analyzed using agarose gel electrophoresis. The gel lanes are labeled as follows: 1, DNA marker (1 kb Extend DNA ladder); 2, ZIKV replicon (15058 bp); 3, DNA marker (1 kb DNA ladder); 4, Fragment 1 (8326 bp); 5, DNA marker (100 bp DNA ladder); 6, Fragment 2 (853 bp); 7, ZIKV mini-rep 1-GFP (8726 bp); 8, ZIKV mini-rep 1-GFP cut by NotI; 9, ZIKV mini-rep 2-GFP (8890 bp); 10, ZIKV mini-rep 2-GFP cut by NotI; 11, ZIKV mini-rep 3-GFP (9868 bp); 12, ZIKV mini-rep 3-GFP cut by NotI.

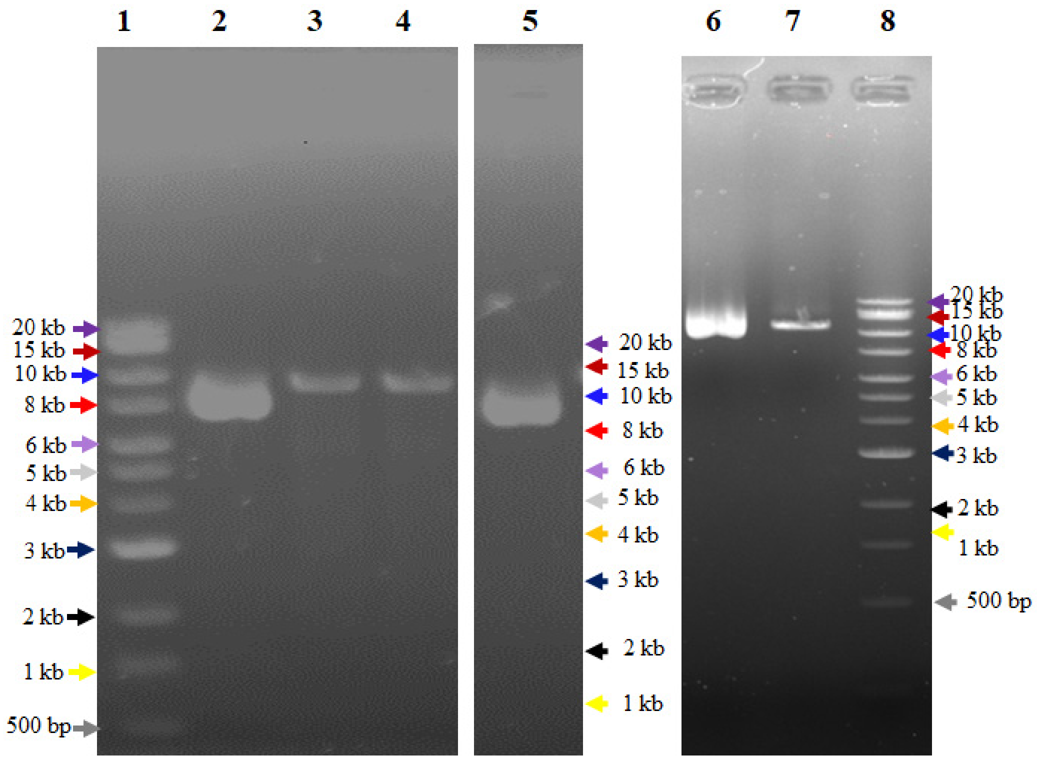

Supplemental Figure 3. Gel electrophoresis analysis of ZIKV mini-replicons containing the CYP reporter (cyan fluorescent protein-linker-yellow fluorescent protein). The sizes of the mini-replicons and cDNA fragment F5 were determined through agarose gel electrophoresis. The gel lanes are labeled as follows: 1, DNA marker (1 kb Extend DNA ladder); 2, Fragment 5; 3, ZIKV mini-rep 2-CYP (9730 bp); 4, ZIKV mini-rep 2-CYP cut by NotI; 5, Fragment 5; 6, ZIKV mini-rep 3-CYP (10708 bp); 7, ZIKV mini-rep 3-CYP cut by NotI; 8, DNA marker (1 kb Extend DNA ladder).
